# Profiles of Maladjustment and Interpersonal Risk Factors in Adolescents

**DOI:** 10.3389/fpsyg.2021.686451

**Published:** 2021-06-22

**Authors:** Inmaculada Méndez, Cecilia Ruiz-Esteban, Gloria Soto, Lucía Granados Alos, Mercedes Matás-Castillo

**Affiliations:** ^1^Department of Evolutionary Developmental and Educational Psychology, Campus Regional Excellence Mare Nostrum, University of Murcia, Murcia, Spain; ^2^Department of Research Methods and Diagnosis in Education, University of Murcia, Murcia, Spain; ^3^Department of Education, Valencian International University, Valencia, Spain

**Keywords:** maladjustment, risk factors, adolescence, mental health, drug

## Abstract

The individual’s adaptation problems can lead to risky behaviors such as drug use. This study aimed to analyze the existence of different adaptation profiles (personal, school, and social) in adolescents. Thus, the study aimed to analyze the existence of significant differences in interpersonal risk factors depending on the degree of adaptation. The study participants were 1,201 students of Compulsory Secondary Education (*M* = 14.43, *SD* = 1.43), and 50.6% were girls. The TAMAI Test (multifactorial adaptation self-evaluation test) and the FRIDA questionnaire (Interpersonal Risk Factors for Drug Use in Adolescence) were used. A latent class analysis (LCA) revealed three different types of adaptation: maladjusted group, at-risk group, and adjusted group. The results showed the existence of significant differences between the different adaptation profiles based on interpersonal risk factors. The data obtained will help school and mental health plans to prevent misbehaving or risky behaviors.

## Introduction

Drug use is a topic of concern in educational groups, as it is a period when adolescents are at great risk of problematic substance use (Rial et al., 2020). The Spanish Observatory on Drugs and Addictions (2020) showed that the drugs most consumed by young people were alcohol and tobacco, followed by cannabis and hypnosedants with or without a prescription. Data binge drinking among adolescents remains a worry. It is therefore necessary to address the tools for both prevention (Fernández-Castillo et al., 2020) and intervention (Vega-González and Pérez, 2021).

One risk factor associated with substance use is social, family, and personal maladjustment (Rueda Aguilar, 2020), which is also linked to school failure (Méndez and Cerezo, 2018). Numerous studies have empirically proven the relationship between social, family, personal, and school factors, which can increase drug use in adolescents (Cerezo et al., 2013; González and Londoño, 2017; Alonso-Castillo et al., 2018; Riquelme et al., 2018; Fernández et al., 2020). Variables such as self-concept, empathy, mood, and violent behavior, among others, are risk factors for consuming substances (Rueda Aguilar, 2020).

There are multiple predictive factors of family maladjustment, including situations of family conflict (hostility, climate of conflict), a negative family climate (stress, negativity, rejection), overprotection and a lack of adequate communication, family relationship quality, and overburdening of families (Pérez de Albéniz-Garrote et al., 2018; Rueda Aguilar, 2020). Moreover, impulsivity, a lack of control (Espinosa and León, 2017), permissive, authoritarian, ambiguous, or inconsistent parenting styles that employ in-process responses to situations of disobedience, a lack of intrafamily communication (Bonnaire and Phan, 2017; Moreno et al., 2020), environments with few rules, and insufficient monitoring by an adult figure (Calero-Plaza et al., 2020) favor adolescent maladjustment in the family context. These factors cause young people who do not know how to manifest problems to react to violence or criminal behavior (Ruiz-Hernández et al., 2019) such as substance use (Rial et al., 2019). By contrast, a positive family relationship, with strong emotional development and family cohesion, will reinforce a good family adaptation that will help prevent situations of social irrigation and therefore drug use in adolescents (Mateo-Crisóstomo et al., 2018; Simón-Saiz et al., 2018). Rial et al. (2019) confirmed that some personal variables such as self-esteem have little impact on cannabis use, compared to family variables such as educating with standards and limits.

When studying adaptation, it is important to note that family adaptation can be a reciprocal factor of good social adaptation. Therefore, family dysfunction and poor parental supervision can incite antisocial and criminal behaviors (Hoeve et al., 2009) that cause social maladaptation and promote drug use (Becoña et al., 2012). In fact, drug use in the group environment is recurrent in adolescents, as they seek to feel socially adapted and link to the peer group (Rueda Aguilar, 2020). From this perspective, social maladaptation is characterized by antisocial behaviors, social mismatch, patterns of criminal behavior (Galinari et al., 2020; Méndez et al., 2021), contexts with few social norms, and even violent norms (Calero-Plaza et al., 2020). To prevent social maladaptation, it is important to enhance influential factors, such as those of the community or neighborhood and those of the surrounding environment (Cutrín et al., 2019; Méndez et al., 2021), and know how to identify group values and attitudes (Rueda Aguilar, 2020). It is also important to recall that various works on social maladaptation have confirmed that a greater incidence of both antisocial and more violent behaviors (Díaz and Moral, 2018; Teixeira and Iossi, 2019; Calero-Plaza et al., 2020) predicts an increased risk of drug use. Moreover, family aspects, such as not feeling supported or recognized by friends or peer groups, can be the cause of greater social maladjustment (Fernández et al., 2020), while social support is considered a key contextual variable for the prevention of social mismatch (Fernández et al., 2020).

Another of the concepts to be highlighted is that of school adaptation, understood as educational achievement (Mortimer et al., 2017) or success in the realization of academic, intellectual, and social factors tasks in school (Rodríguez-Fernández et al., 2016). With respect to school adaptation, factors that can propel maladjustment include poor academic performance, environmental factors such as little social support (Moreira et al., 2018), poor qualifications, a lack of academic self-efficacy, responsibility or commitment to school (Pelegrín and Garcés de Los Fayos, 2009), the influence of classmates (Mikami et al., 2017), poor teaching and family support (Fernández et al., 2020), being a repeater (Méndez and Cerezo, 2018), and violence (Calero-Plaza et al., 2020; Méndez et al., 2021). A diversity of interconnected variables is evidenced that influence social adaptation (Terrón and Hurtado, 2020) and affect one another. Therefore, managing stressful situations requires the consideration of strategies that benefit academic performance, such as support, learning skills, motivation to study, and school self-concept. These variables that encompass good school adaptation can result in good school performance (Fernández et al., 2020; Valiente-Barroso et al., 2020). In addition, it should be noted that school adaptation is linked to behavioral (such as participation in aspects of the school), emotional (such as feeling recognized or belonging to the school), and cognitive (such as actively participating in the tasks of the teaching and learning process) variables (Fredricks et al., 2004; Rodríguez-Fernández et al., 2018). On the other hand, although social maladjustment is linked to family maladjustment factors, in the case of school maladjustment, there is also a relationship between good family and social adaptation and adaptation in school (Pérez-Fuentes et al., 2015; Rodríguez-Fernández et al., 2018; Fernández et al., 2019). Furthermore, adolescents who consider that they have greater support from their social and family environments will show better academic progress.

Another important aspect of this work is the concept of personal maladjustment. Personal maladjustment is undoubtedly influenced by situations of family, social, and school maladjustment. Variables include personal dissatisfaction and affective maladjustment, psychological factors (Molero et al., 2017; Mayorga et al., 2020), self-devaluation, anxiety, hypersensitivity, guilt, pessimism (Palacio et al., 2017), emotional variables, mood, and self-perception, among others (Simón-Saiz et al., 2018). Low self-concept is one of the most important variables when discussing personal maladjustment, as it plays a decisive role in students’ personal adjustment and school maladjustment (Fernández et al., 2019) as well as in their decisions. It is also associated with cognitive–behavioral variables (finding better levels of adaptation if the student presents attitudes to review thoughts and actions to deal with the novelty) and emotional (where more adapted students show skills for reducing negative emotions such as fear or frustration in uncertain situations; Zhang et al., 2020).

Finally, it can be emphasized that the different forms of maladjustment are interrelated: Good personal adaptation can be the cause and consequence of good family adaptation. Furthermore, social adaptation can cause family and personal maladjustment, and vice versa (Fernández et al., 2019). If the relationship between the different types of maladjustment is mutual, it is important to work on the three dimensions, because the more resources adolescents have, the less drug use there will be. As a result, adolescents who are not drug users present more personal resources and better psychosocial adjustment, aspects that are reflected by greater stability in family relationships and an appreciation of their family context as united and affectionate and as a place where they can express themselves freely. This, in turn, helps families to advise and support adolescents in problematic situations of substance use (Cerezo et al., 2013; Rueda Aguilar, 2020). For adolescents, at the social level, having more resources is manifested by greater social adaptation, while having fewer social skills can be associated with greater consumption of alcohol and drugs. Personal maladjustment variables, such as levels of depression, loneliness, and unhappiness, have been understood as risk factors for substance use (Espada et al., 2018). Low self-control and emotional problems have also been linked to substance use (Oliva et al., 2019; Sánchez et al., 2019).

Given the aforementioned, this study aimed to analyze the existence of different adaptation groups (personal, school, and social) in adolescents and significant differences in interpersonal risk factors depending on the degree of adaptation.

## Materials and Methods

### Participants

The study participants were 1,201 students of Compulsory Secondary Education from different geographical areas of the region of Murcia in Spain (44.6% first and second course and 55.4% third and fourth course), aged 11–18 years (*M* = 14.43, *SD* = 1.43); 50.6% were girls. The students belonged to public centers (65.8%) and private/semi-private centers (34.2%). The distribution was homogeneous in terms of gender and course (*χ*^2^ = 5.70, *p* = 0.13). In the same way, the distribution was homogeneous in terms of gender and age (*χ*^2^ = 0.42, *p* = 0.81). Therefore, 13.5% were girls aged 11–13, 25.6% were girls aged 14–15, and 11.5% were girls aged 16–18 (see Table 1). A total of 35.6% had repeated some school year. The socioeconomic level of the areas and schools was average.

**Table 1:** Sample distribution according to age and gender.

Variables	Age	11–13 age	14–15 age	16–18 age
Gender	Boys	164 (14%)	287 (24.4%)	129 (11%)
	Grils	158 (13.5%)	301 (25.6%)	135 (11.5%)

#### Design and Procedure

The Ethics Committee of the University of Murcia (ID: 2478/2019) approved the study protocol. The data of the present work were collected as part of a larger project focused on the analysis of the intra- and interpersonal variables that influence teaching-learning. It is a descriptive cross-sectional study. The study participants were selected students from Compulsory Secondary Education in the region of Murcia, Spain. For data collection, first of all, a telephone contact was made with the school principals, followed by a meeting with the principals and school psychologists jointly from the participating schools in order to present the objectives and purpose of the research, describe the assessment instruments, and request their permission and collaboration. To encourage their cooperation, they were also told that a final report with the results of the study with result-based guidance would be provided to each center to enable measures to be applied in each school.

It was necessary to have permits and school collaboration and to obtain the informed consent of all the participants and their parents. The study instruments were administered in the classrooms of the schools in a 50-min session. Anonymity, voluntariness, and confidentiality were maintained at all times.

### Instruments

Three Evaluation Instruments Were Used for the Study.

First, we used a sociodemographic questionnaire that measured gender (male/female), age, course repetition (yes/no), type of school (public/private/semi-private), and country of birth.

We used the FRIDA Test—Interpersonal Risk Factors for Drug Use in Adolescence (Secades et al., 2006), consisting of 90 Likert scale-type (3 or 5 points) questions with the following factors: family reaction to drug use (refers to the reaction of different members of the family to a possible use of legal or illegal drugs; thus, high values indicate a greater risk in the family for consumption), group of friends (refers to the attitude of the group of friends toward the consumption as well as the activities that the friends carry out related to drug use; thus, high values indicate a group of friends with positive attitudes toward drugs and with risky actions toward drugs), access to drugs (refers to students’ perception of the ease of accessing drugs; thus, high values indicate a greater perception of access to drugs), family risks (refers to drug use and family problems; thus, the highest values indicate the existence of family conflicts and drug use in the family environment), family education on drugs (measures whether the family has given preventive educational guidelines against drug use; thus, high values indicate the lack of family education about the risks and consequences of drugs), protective activities (refers to quality family relationships, school variables that can protect against drug use; thus, high values indicate the lack of protection activities against drug use in leisure and free time, in schools, etc.), and educational style (refers to the parents’ educational style; thus, high values indicate permissive educational styles). Similarly, another factor in the degree of global vulnerability is presented by the adolescent. The direct scores obtained in each factor, as well as the global index, are transformed into a scale (Secades et al., 2006). Therefore, the transformed scores indicate the degree of vulnerability that the subject presents. The whole scale had a Cronbach’s α reliability index of 0.92, being 0.93 according to Secades et al. (2006). Example of item: Drugs are used in my family.

Second, we used the multifactorial adaptation self-evaluation test (TAMAI; Hernández-Guanir, 2015), which consisted of 175 dichotomous items assessing the following factors: personal maladjustment (indicative of the person’s level of mismatch with themselves and the general reality or personal difficulty in accepting reality as it is), school maladjustment (refers to dissatisfaction and inappropriate behavior with respect to school reality, can manifest as: unfavorable attitudes toward learning, low school achievement, disruptive behaviors, etc.), and social maladjustment (indicates a disability or difficulties in social relationships, showing a lack of compliance with social norms, social distrust relationships, lack of social control, lack of respect for or consideration of others, etc). The test has a Cronbach’s alpha of 0.92, and it was 0.94 in our study. Example of item: I am unruly and disobedient.

### Data Analysis

The LCA was estimated in the first place to classify the participants according to the scores obtained in adaptation (TAMAI). The LCA was used because it is a precise technique that allows for overcoming the limitations with the grouping of K-means (Schreiber, 2017). The Bayesian Information Criterion (BIC) and entropy values were used to evaluate the fit of the model and especially to determine the most adequate number of latent classes. Literature has shown that lower BIC and Akaike Information Criterion (AIC) values are the most adequate fit indices for choosing the best class solution (Smeets et al., 2017). In the same way, the viability theory was used together with the psychological meaning of each of the groups that represented the different adaptation profiles and that therefore maximized the differences between the classes. Therefore, the groups of students were defined based on three types of different degrees of adaptation (TAMAI) attending to the risk levels according to the values obtained in the scores transformed in the FRIDA: (a) high personal, school, and social maladjustment; (b) moderate personal, school, and social maladjustment; and (c) low personal, school, and social maladjustment. An analysis of variance (ANOVA) was performed, using *post hoc* tests with the Bonferroni method to analyze the differences between the groups and the different adaptation groups (personal, school, and social) according to the dimensions of the risk factor interpersonal skills for drug use. It was necessary to estimate partial eta-squared (*ηp*^2^) as well as Cohen’s d (1998) to determine the magnitude of the differences. We, therefore, used SSPS Statistics (version 23.0) and the Excel package (XLSTAT).

## Results

In Table 2, see descriptive statistics and Pearson’s correlation between the FRIDA and the TAMAI subscales. They were mostly significant and positive so the LCA was effected.

**Table 2:** Descriptive statistics and bivariate correlations between the FRIDA and the TAMAI subscales.

Variable	Personal maladjustment	School maladjustment	Social maladjustment	*M*	*SD*
Personal maladjustment	--	--	--	7.52	5.66
School maladjustment	0.349^∗∗∗^	1	--	11.58	6.97
Social maladjustment	0.548^∗∗∗^	0.562^∗∗∗^	--	8.50	5.02
Family reaction	0.135^∗∗∗^	0.294^∗∗∗^	0.216^∗∗∗^	26.82	11.66
Peers	0.154^∗∗∗^	0.354^∗∗∗^	0.223^∗∗∗^	19.79	5.46
Access to drugs	−0.005	−0.091^∗∗^	−0.036^∗∗∗^	23.32	10.08
Family risks	0.251^∗∗∗^	0.279^∗∗∗^	0.265^∗∗∗^	25.86	6.48
Family education	0.151^∗∗∗^	0.164^∗∗∗^	0.175^∗∗∗^	16.33	5.20
Protective activities	0.180^∗∗∗^	0.314^∗∗∗^	0.260^∗∗∗^	47.50	10.85
Educational styles	0.136^∗∗∗^	0.297^∗∗∗^	0.225^∗∗∗^	20	8.11
Global vulnerability	0.211^∗∗^	0.348^∗∗∗^	0.287^∗∗∗^	179.62	37.13

∗∗ *p* < 0.01;

∗∗∗ *p* < 0.01.

**Figure 1:**
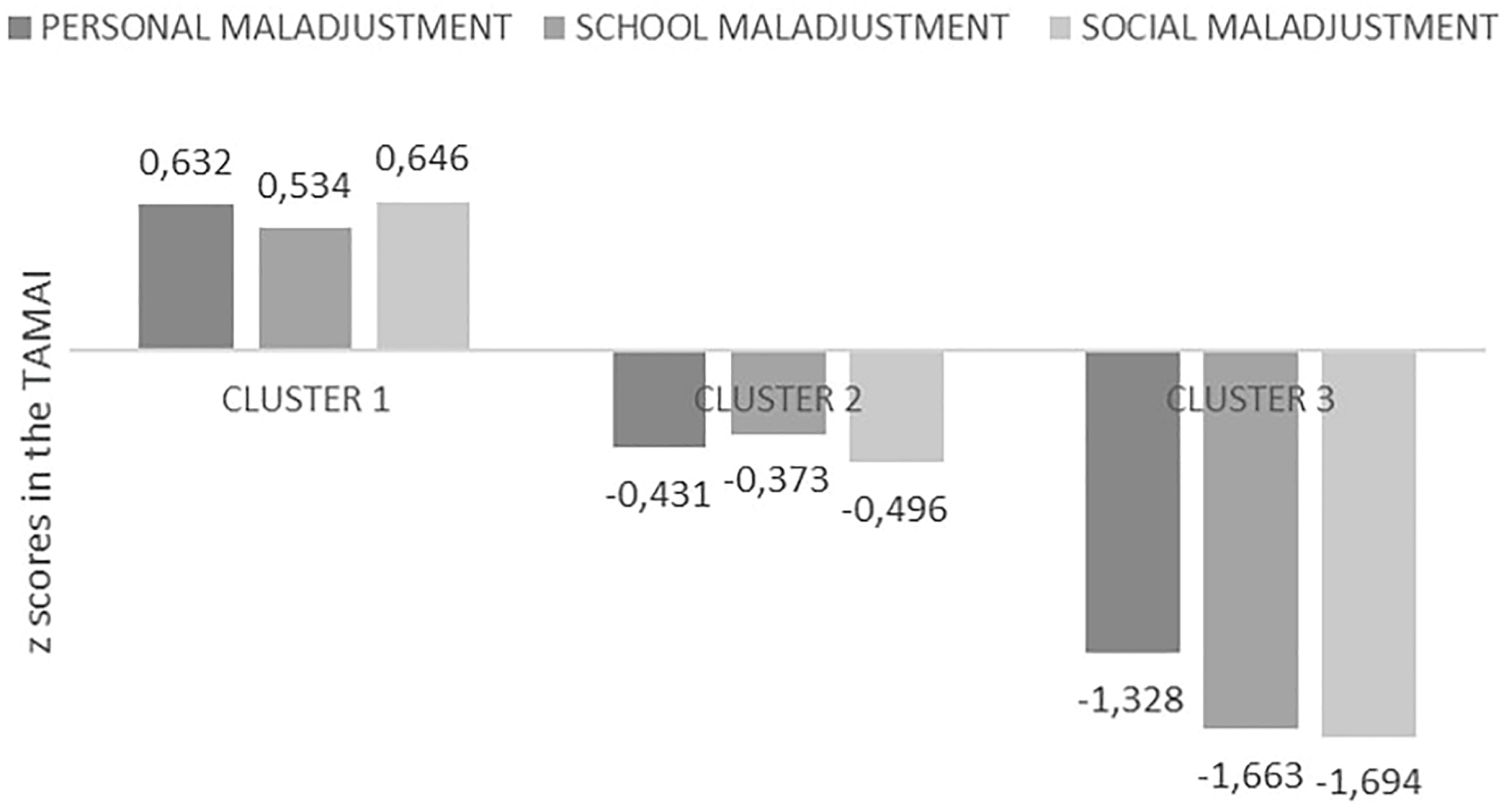
Graphical representation of the three-cluster model.

Table 3 presents the models obtained (from two to four clusters). Model 3 presents the best and the less BIC and the AIC values, which is why it was selected. The LCA identified three different types of adaptation: (a) high personal, school, and social maladjustment (maladjusted group); (b) moderate personal, school, and social maladjustment (at-risk group); (c) low personal, school, and social maladjustment (adjusted group; see Figure 1).

**Table 3:** The fit of the all latent class models.

Clusters	BIC	AIC	Entropy
2	9,492,709	9,426,528	0.706
**3**	**8,260,090**	**8,202,257**	**0.742**
4	8,339,712	8,358,272	0.651

BIC, Bayesian Information Criterion; AIC, Akaike Information Criterion. Values in bold show the selected model.

Table 4 reports on the absolute and relative percentage of students in each class of the best model as well as the accuracy of classification in each class. Class 1 was made up of 581 students (48.4%), class 2 was made up of 546 students (45.4%), and class 3 was made up of 74 students (6.2%). With respect to the probabilities of belonging to each class, Table 4 shows that the most precise class is class 3, which has a precision of 99.9% (see the diagonals of the table), followed by class 1, which correctly classifies 85.9% of the students.

**Table 4:** Frequency of students in the classes and accuracy of classification in each class of the best model.

Classes	af	rf	Class 1	Class 2	Class 3
1	581	0.484	0.859	0.141	0.000
2	546	0.454	0.160	0.839	0.001
3	74	0.062	0.000	0.001	0.999

af, absolute frequency; rf, relative frequency.

The ANOVAs showed significant differences between the three groups of adaptation according to the dimensions of interpersonal risk factors (see Table 5).

**Table 5:** Means and standard deviations obtained by the three groups of adaptation and values of the partial eta-squared (*η_p_*^2^) for each dimension of interpersonal risk factors.

	Group 1		Group 2		Group 3		Significance		
Dimensions	*M*	*SD*	*M*	*SD*	*M*	*SD*	*F_(2,1,198)_*	*p*	*η_p_*^2^
Family reaction	28.88	11.31	26.07	10.25	16.19	16.70	43.91	<0.001	0.068
Peers	20.91	4.51	19.67	4.26	11.96	10.90	103.78	<0.001	0.148
Access to drugs	22.55	8.99	25.36	9.59	14.36	14.89	45.14	<0.001	0.070
Family risks	27.34	5.10	25.74	4.46	15.09	14.17	145.33	<0.001	0.195
Family education	16.99	4.50	16.62	4.34	8.88	9.02	94.39	<0.001	0.136
Protective activities	49.41	7.35	48.58	6.86	24.51	24.06	252.35	<0.01	0.296
Educational styles	21.43	7.59	19.93	7.20	9.24	10.06	84.41	<0.01	0.124
Global vulnerability	187.51	22.62	181.97	22.18	100.24	89.45	263.47	<0.01	0.305

Cluster 1 (maladjusted), Cluster 2 (at-risk), and Cluster 3 (adjusted).

In Table 6, see Cohen’s d indices for the *post hoc* contrast groups and the differences that were found to be significant between the groups.

**Table 6:** Cohen’s d indexes for *post hoc* contrast groups.

Dimensions	Group1–Group 2	Group1–Group 3	Group 2–Group 3
Family reaction	---	---	---
Peers	0.26^∗∗∗^	1.05^∗∗∗^	0.88^∗∗∗^
Access to drugs	0.28^∗∗∗^	1.60^∗∗∗^	1.41^∗∗∗^
Family risks	0.33^∗∗∗^	0.83^∗∗∗^	1.06^∗∗∗^
Family education	n.s.	1.81^∗∗∗^	1.66^∗∗∗^
Protective activities	n.s.	1.56^∗∗∗^	1.51^∗∗∗^
Educational styles	---	2.35^∗^	2.30^∗∗∗^
Global vulnerability	0.20^∗∗∗^	1.54^∗∗∗^	1.41^∗∗∗^

∗ *p* < 0.05;

∗∗∗ *p* < 0.001.

n.s., not significance.

*Post hoc* comparisons reported that the maladjusted group had higher values than the adjusted group for all risk factors for drug use (group of friends, access to drugs, family risks, family education on drugs, protective activities, educational style, and global vulnerability) except for the family reaction to drug use, which was not significant.

Similarly, the maladjusted group presented higher values than the at-risk group in a group of friends, family risk, and global vulnerability. However, the at-risk group presented high values in ease of access to drugs in contrast to the maladjusted group. Family reaction to drug use, family education on drugs, protective activities, and educational styles were not significant.

On the other hand, the at-risk group presented higher values than the adjusted group in all risk factors for drug use (group of friends, access to drugs, family risks, family education on drugs, protective activities, educational style, and global vulnerability). However, differences were not found in the family’s reaction to drug use.

## Discussion

This study made it possible to pursue the general objective of analyzing the existence of different adaptation groups in adolescents and of verifying the link between risk factors for drug use. As a result, the existence of three adaptation groups was evidenced: a high-risk profile or maladjusted group, a moderate risk profile or risk group, and a low-risk or adjusted group profile.

In this study, the LCA identified three different types of adjustment in the adolescent.

Similar to our study, other studies identify three groups of students based on interpersonal values (Gázquez et al., 2015a,b, 2016) or three adjustment groups (Spilsbury et al., 2008; McDonald et al., 2016; Sianko et al., 2016).

The results of the study confirmed that the maladjusted group had a higher maladjustment profile compared to the adjusted group, which agrees with studies such as those by Sánchez et al. (2019) and Oliva et al. (2019), who pointed out that situations of imbalance incite drug use in adolescent. Thus, the maladjusted group presented higher values than the adjusted group for all risk factors for drug use (group of friends, access to drugs, family risks, family education on drugs, protective activities, educational style, and global vulnerability; Rueda Aguilar, 2020). A group of friends with a high level of drug use is a variable that should be valued with great consideration because the peer group has high relevance in adolescence (Cerezo et al., 2013; Teixeira and Iossi, 2019; Fernández et al., 2020). Many adolescents consume substances because they feel more adapted to the group, and if the group uses, the risk factor increases which may be associated with a greater perception of access to drugs (Alfonso et al., 2009; Pérez-Fuentes et al., 2015; Rueda Aguilar, 2020; Méndez et al., 2021). High values were also reached in family factors, such as the family risk of drug use; low family education on drugs; and therefore, a lack of norms concerning drug use, a permissive educational style, a lack of protective activities in the family, and family relationships (Pérez-Fuentes et al., 2015; Calero-Plaza et al., 2020; Moreno et al., 2020; Momeñe et al., 2021). In addition, the maladjusted group obtained higher scores in a higher level of global vulnerability to drug use (Alfonso et al., 2009; Fernández et al., 2020). Therefore, the results found in the maladjusted group make it evident that programs to improve said vulnerability are very necessary. Above all, considering the data on the high-risk profile of drug use found in the study and its possible associations with other risk factors, it is important to develop prevention and intervention programs along these lines. Psychoeducational interventions based on promoting strategies that favor self-regulation for good mental, emotional, and behavioral development (Casuso-Holgado et al., 2019; Barroso et al., 2020; Fernández et al., 2020) and promote self-concept in the school context should be worked on or proposed (Valiente-Barroso et al., 2020). Moreover, working on awareness, training, and the participation of families is a very important factor in the development of good adaptation (Pérez-Fuentes et al., 2015; Moreno and Palomar, 2017).

The at-risk group was in a vulnerable situation, as it shared risk factors similar to the maladjusted group and was therefore different from the adjusted groups. This indicated a group of students with a moderate risk of drug use due to the high number of risk factors (group of friends, family risk, and global vulnerability). In addition, the at-risk group presented high values in ease of access to drugs in contrast to the maladjusted group and the adjusted group. This may indicate that the at-risk group was at risk of a possible imbalance and that the perception of the student being able to access drugs if they wanted was high (Alfonso et al., 2009). In the same way, the family can pose a risk for consumption due to risky or conflictive situations, drug use in the family environment, or low family education about the risks of drugs (Pérez-Fuentes et al., 2015; Kuntsche and Kuntsche, 2016; Calero-Plaza et al., 2020; Moreno et al., 2020). Therefore, these results are indicators that this requires action so that the moderate values do not suppose a situation of greater risk and that the student can have maladjusted behaviors and therefore put their health at risk (Cerezo et al., 2013; Molero et al., 2020). Another aspect to work on would be the promotion of emotional aspects (Méndez et al., 2019; Peña-Casares and Aguaded-Ramírez, 2021) that exert a factor of prevention and resolution of situations of imbalance, leading the risk group into substance use, as drug use can be related to the affective dimensions, or to an imbalance in the family context, where an absent parental figure can lead to a consuming descendant (Martínez et al., 2013; Pérez-Fuentes et al., 2015; Espada et al., 2018; Oliva et al., 2019; Sánchez et al., 2019). Therefore, in addition to working on values and promoting emotionality, proposals for the creation and development of safe schools could be established: As pointed out by UNESCO-IICBA (2017), the consolidation of positive environments enhances integral development (physical, social, emotional, and cognitive) in adolescents and increases prevention systems on their health.

The last group discussed is the group of students who presented low personal, school, and social maladjustment (adjusted group). This group had a low risk of drug use and possibly higher levels of adaptation. The adapted presented adequate values in relation to the group of friends, access to drugs and at the family level (low family risk, adequate family education, and low global vulnerability). As mentioned above, good adaptation in one context can be linked to good adaptation in another context. For example, good family adaptation affects good social adaptation (Alonso-Castillo et al., 2018; Riquelme et al., 2018; Fernández et al., 2020). In addition, different studies have concluded that adolescents who do not use drugs generally show more personal resources and better psychosocial adjustment than adolescents who use drugs (Espada et al., 2018; Oliva et al., 2019; Sánchez et al., 2019; Pérez-Fuentes et al., 2019) as well as better social adaptation (Teixeira and Iossi, 2019). This adjusted group may have also shown these low levels of drug use because of aspects such as those pointed out that a good family relationship or a good family context is a protective factor against risk situations in the face of drug use (Alonso-Castillo et al., 2018; Mateo-Crisóstomo et al., 2018; Simón-Saiz et al., 2018).

Given the importance highlighted throughout the work, and having exposed some alternatives to working with adolescents to enhance their adaptation and reduce the chances of using drugs emphasizing that from the school environment, it is necessary to continue building positive educational contexts that generate school commitment or student involvement in school (Pinazo, 2019) and taking into consideration the benefits of promoting norms among peers (Laninga-Wijnen et al., 2021) where the teacher has a fundamental role in promoting school adaptation, implementing activities that promote emotional development (Fernández et al., 2020). From such a vision of educational contexts that promotes integral development, research has increasingly focused on promoting factors such as resilience or self-concept (Rodríguez-Fernández et al., 2018).

This study clarified the need to work on the variables of personal, family, school, and social adaptation, due to their link with maladjusted behaviors, which lead to drug use. This coincides with numerous studies that confirm this cause-and-effect relationship between maladjustment and variables that affect adaptation and substance abuse (Espada et al., 2018; Rueda Aguilar, 2020).

To conclude, we want to highlight that this study provides information on the adaptation profiles and risk of drug use, by giving an overview of some variables that influence this concept. A limitation of this study is that it has been a cross-sectional study focused on the use of questionnaires for students, so it would be appropriate to expand the information through interviews and even through longitudinal studies. It would also be advisable to have the opinion of the family and teachers about the perception of consumption by students. In the same way, it is necessary to develop future research to delve more deeply into different variables that were not taken into account in this research, such as family factors, including parenting styles and their relationship to their children’s adaptation and situations of drug abuse, repeater schoolchildren, and more personal aspects, such as self-concept or resilience.

Finally, it should be noted that despite the stated limitations, the present work highlights the importance of using protective factors and employing different types of strategies such as personal, family, and social aspects. And above all, the approach of working on all these factors is through educational programs for the prevention of substance abuse in adolescents.

## Data Availability Statement

The raw data supporting the conclusions of this article will be made available by the authors, without undue reservation.

## Ethics Statement

The Ethics Committee of the University of Murcia (ID: 2478/2019) approved the study protocol. It was necessary to have permits and school collaboration and to obtain the informed consent of all the participants and their parents. The study instruments were administered in the classrooms of the schools in a 50-min session. Anonymity, voluntariness, and confidentiality were maintained at all times.

## Author Contributions

IM, GS, and CRE contributed to the conception and design of the review. IM, MMC, and LGA applied the search strategy. IM, CRE, GS, LGA, and MMC applied the selection criteria, completed the bias-risk assessment, and analyzed and interpreted the data. All authors contributed to the article and approved the submitted version.

### Conflict of Interest

The authors declare that the research was conducted in the absence of any commercial or financial relationships that could be construed as a potential conflict of interest.
